# Point-of-Care Quantification of Serum Alpha-Fetoprotein for Screening Birth Defects in Resource-Limited Settings: Proof-of-Concept Study

**DOI:** 10.2196/23527

**Published:** 2020-08-14

**Authors:** Balaji Srinivasan, Julia L Finkelstein, David Erickson, Saurabh Mehta

**Affiliations:** 1 Division of Nutritional Sciences, Cornell University, Ithaca, NY, United States; 2 Sibley School of Mechanical and Aerospace Engineering, Cornell University, Ithaca, NY, United States

**Keywords:** alpha-fetoprotein, point-of-care testing, screening, neural tube defects, mobile phone

## Abstract

**Background:**

Maternal serum alpha-fetoprotein (MSAFP) concentration typically increases during pregnancy and is routinely measured during the second trimester as a part of screening for fetal neural tube defects and Down syndrome. However, most pregnancy screening tests are not available in the settings they are needed the most. A mobile device–enabled technology based on MSAFP for screening birth defects could enable the rapid screening and triage of high-risk pregnancies, especially where maternal serum screening and fetal ultrasound scan facilities are not easily accessible. Shifting the approach from clinic- and laboratory-dependent care to a mobile platform based on our point-of-care approach will enable translation to resource-limited settings and the global health care market.

**Objective:**

The objective of this study is to develop and perform proof-of-concept testing of a lateral flow immunoassay on a mobile platform for rapid, point-of-care quantification of serum alpha-fetoprotein (AFP) levels, from a drop of human serum, within a few minutes.

**Methods:**

The development of the immunoassay involved the selection of commercially available antibodies and optimization of their concentrations by an iterative method to achieve the required detection limits. We compared the performance of our method with that of commercially obtained human serum samples, with known AFP concentrations quantified by the Abbott ARCHITECT chemiluminescent magnetic microparticle immunoassay (CMIA).

**Results:**

We tested commercially obtained serum samples (N=20) with concentrations ranging from 2.2 to 446 ng/mL to compare the results of our point-of-care assay with results from the Abbott ARCHITECT CMIA. A correlation of 0.98 (*P*<.001) was observed on preliminary testing and comparison with the CMIA. The detection range of our point-of-care assay covers the range of maternal serum AFP levels observed during pregnancy.

**Conclusions:**

The preliminary test results from the AFP test on the mobile platform performed in this study represent a proof of concept that will pave the way for our future work focused on developing a mobile device–enabled quad-screen point-of-care testing with the potential to enable the screening of high-risk pregnancies in various settings. The AFP test on the mobile platform can be applied to enable screening for high-risk pregnancies, within a few minutes, at the point of care even in remote areas where maternal serum tests and fetal ultrasound scans are not easily accessible; assessment of whether clinical follow-up and diagnostic testing may be needed after a positive initial screening evaluation; and development of surveillance tools for birth defects.

## Introduction

### Background

Neural tube defects (NTDs) are one of the most common and debilitating birth defects documented in the United States and globally. These defects arise when the neural folds fail to fuse entirely during early embryogenesis. Outcomes for an infant born with an NTD vary by severity and the affected region of the neural fold [1,2]. Anencephaly and other open NTDs that affect the infant’s brain are incompatible with life, further leading to fetal loss during pregnancy or death soon after birth, whereas those affecting the spine can lead to serious neurological and physical impairment [1,2]. Closed NTDs have a layer of skin covering the defect and are less severe; however, they can require surgery and cause motor and sensory impairments [1,2]. It is estimated that there are over 260,000 NTD cases globally per year, with the burden ranging from 1 to 80 per 10,000 births globally, leading to 70,800 deaths and loss of 6.4 million disability-adjusted life years [3–8]. The total lifetime direct cost of care [9] for a child born with spina bifida in the United States is estimated to be US $791,900.

In cases with elevated maternal serum alpha-fetoprotein (MSAFP) levels, an ultrasound examination is recommended to further determine whether an NTD or another anomaly associated with elevated MSAFP levels is present, in addition to confirming the gestational age, fetal viability, and number of fetuses. If ultrasound findings are ambiguous or show an apparently normal fetus, then genetic counseling and further evaluation through amniocentesis are usually performed. Screening and an early diagnosis of affected pregnancies provides parents with the options for diagnostic and clinical follow-up, interventions during pregnancy, and preparation for the birth of an affected child, including associated medical costs for surgical or nonsurgical treatments. In addition, prenatal NTD detection also informs clinical triage regarding the optimal timing, route, and site of delivery (eg, referral to high-risk or tertiary care hospitals, cesarean delivery). In the United States, the universal screening for NTDs is supported by the American College of Obstetricians and Gynecologists (ACOG) [10] and the American College of Medical Genetics [11], while emphasizing the need to provide adequate counseling and follow-up services. NTD screening approaches, such as biomarker assessment (measurement of MSAFP) and ultrasound examinations (anatomical), also enable the screening and detection of fetal abnormalities other than NTDs and inform clinical care and follow-up [12,13].

MSAFP is one of the biomarkers included in the triple screen test for pregnant women. The triple screen test is a maternal blood screening test that looks for 3 distinct analytes—MSAFP, human chorionic gonadotropin (hCG), and unconjugated estriol—to identify women who are at an increased risk of having a baby with NTD or trisomy syndrome. The triple screening is recommended between 15 and 22 weeks of gestation and is most accurate if performed between 16 and 18 weeks of gestation. Fetal serum alpha-fetoprotein (AFP) concentrations peak at 10–13 weeks’ gestation and decline progressively until term, whereas maternal levels peak during the third trimester [14]. Elevated MSAFP levels with a screen positive rate of 5% or less can detect 75%−90% of NTDs and ≥95% of anencephaly [11], which is the most severe type of NTD that is incompatible with life [1]. Abnormally low AFP values [15] (most often a median value of <0.5) are associated with Down syndrome and other chromosomal abnormalities. MSAFP levels may also detect 85% of the ventral wall defects [11].

MSAFP levels are typically quantified using immunoassay-based methods. Conventional immunoassays include enzyme-linked immunosorbent assay [16], radioimmunoassay [17], fluoroimmunoassay [18], electrochemiluminescence [19], and the latex-enhanced immunoturbidimetric method [20]. Several fully automated benchtop instruments, such as the μTasWako i30 (Fujifilm Wako Diagnostics), IMMULITE 2000 Xpi Immunoassay system (Siemens Healthineers), and the ARCHITECT *i*1000SR immunoassay analyzer (Abbott Diagnostics), are also commercially available. However, many settings do not have access to cold chain and centralized laboratories for these laboratory tests. Typically, these conventional immunoassays take a few days, starting from sample collection to a patient finally getting access to test results through a health care provider.

Mobile platforms (smartphones) are positioned to be the hub of the future of medicine [21], with smartphone- and tablet-based medical devices continuing to be integrated into patients’ lives in various settings. The increased use of smartphone-based apps and analytical devices has been demonstrated in recent years for numerous apps such as diet tracking apps [22], well-being apps [23], environmental monitoring [24], food toxin screening [25], and medical diagnostics [26–29]. Point-of-care testing (POCT), using smartphones [30], is rapidly emerging as a potential alternative to conventional screening and laboratory-based diagnostic testing, particularly in resource-limited settings.

### Objectives

In this study, we present a proof of concept for the lateral flow immunoassay–based rapid screening of serum AFP levels on a mobile platform, from a drop of human serum, within a few minutes. We aim to demonstrate the quantification of AFP in commercial serum calibrators and preliminary results with commercially obtained human serum samples with known AFP concentrations, quantified by the Abbott ARCHITECT CMIA. Preliminary results from this work will pave the way for our future work focused on developing a mobile device–enabled quad-screen test at the point of care in resource-limited settings.

## Methods

### Overview

The components of the test strip were selected to achieve optimum flow rates as well as the volume of reagents and AFP in the test samples. Development of the immunoassay involved the selection of commercially available antibodies and optimization of their concentrations by an iterative method to achieve the required detection limits. The entire testing process is guided by a mobile app *AFPhone*, which is designed to input important patient information, provide step-by-step instructions to the user for running the test, and display the AFP concentrations on the device screen. Briefly, the testing process required a drop of the test sample on the test strip to initiate the test. The camera within the portable reader captures the relative intensity changes of the colored bands on the test strip. A custom code is used to batch process the captured images of the test strip and provide the AFP concentrations. The test strip design was optimized, and the calibration curves were experimentally determined using commercially available serum-based AFP calibrators and commercially obtained serum samples with known AFP concentrations. This study does not contain any studies involving human participants; hence, ethical approval was not required. This development process is described in detail in the following sections.

### Reagents and Materials

Gold nanoparticle (AuNP) conjugation kit (InnovaCoat 20OD) with 40 nm diameter AuNPs were obtained from Expedeon, Inc. Purified AFP from human fetal cord serum (Cat# 8F8) and monoclonal mouse antihuman-AFP antibodies (Hytest Cat# 4F16–4A3, RRID:AB_2223930, and Hytest Cat# 4F16–5H7, RRID:AB_2223929) were purchased from HyTest Ltd (Finland). Rabbit antimouse immunoglobulin G (IgG) was purchased from Jackson ImmunoResearch Inc. Audit AFP calibrators (Linearity FD Tumor Markers, Cat# K719M-5) were purchased from AUDIT MicroControls, Inc. Amine-free phosphate buffer saline (at 0.01 M) with a pH of 7.4, Tween 20, bovine serum albumin (BSA), borate buffer, and sucrose were acquired from Sigma-Aldrich. A glass fiber conjugate pad with dimensions of 300 mm × 10 mm, Hi-Flow Plus 180 membrane cards, and a cellulose fiber pad for an absorbent pad were acquired from EMD Millipore. The membrane for the sample pad was purchased from mdi Membrane Technologies, Inc.

### Equipment

The following equipment was used in this study: lateral flow reagent dispenser (Claremont BioSolutions, Upland), Legato 200 Dual Syringe Pump (Claremont BioSolutions LLC), matrix 2360 programmable shear (Kinematic Automation, Inc), and V-1200 Spectrophotometer.

### Technology and Components

**Figure 1.**
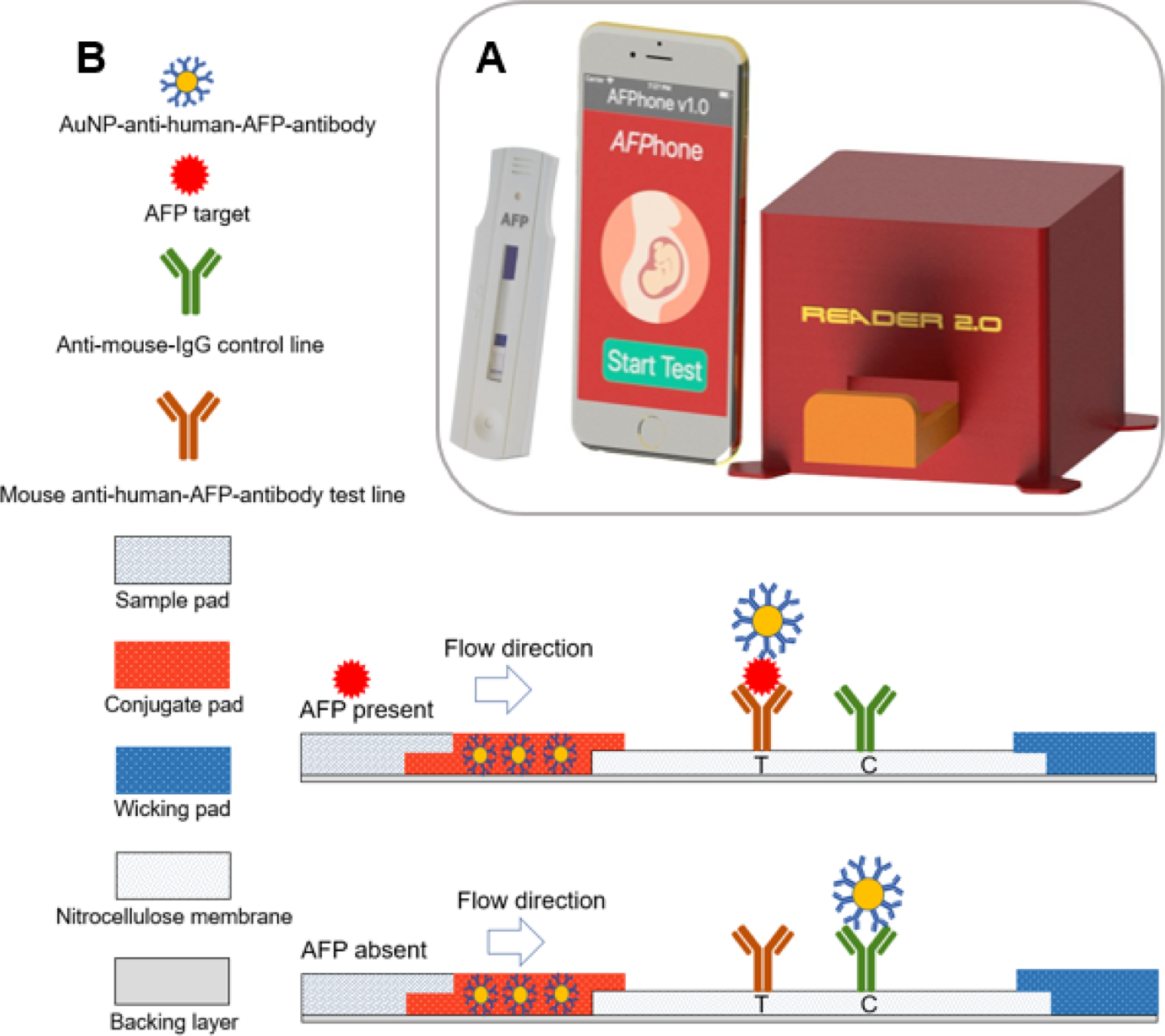
(A) Components of the point-of-care testing system. (B) Schematic showing various. components of the test strip with sandwich-type assay for alpha-fetoprotein detection. AFP: alpha-fetoprotein; AuNP: gold nanoparticle; C: control; IgG: immunoglobulin G; T: test.

The technology comprises a custom-made test AFP test strip, cassette for housing the test strip, portable test strip reader, and a mobile app *AFPhone* to guide the user through the various steps of the testing protocol. A custom-developed image processing code was applied to batch process the acquired images to compute the test and control line intensity (TC) ratios for each test strip. Figure 1 shows the technology components of the point-of-care approach described in this study.

### Test Strip Architecture and Immunoassay Format

The AFP test strip in Figure 1 was based on a sandwich format immunoassay and comprised a whole blood filtration membrane as the sample pad; a conjugate pad for prestoring the AuNP–antihuman–AFP–antibody conjugates in a dry form; a nitrocellulose membrane with antihuman–AFP monoclonal antibodies and secondary antibodies, respectively; and a wicking pad made of cellulose fiber that functions as a waste reservoir. The addition of the test sample and running buffer causes the AuNP–antihuman–AFP-antibody conjugates to flow freely due to capillary action and react with AFP in the test sample. At high AFP concentrations in the test sample, most of the AuNP–antihuman–AFP–antibody conjugates will bind with the free AFP, eventually binding to the antihuman–AFP–antibody on the test line, resulting in a sandwich complex. All unbound AuNPs–antihuman–AFP were captured at the control line. This relative binding of the AuNP–antihuman–AFP–antibody at the test and control lines increases the test line (T) to control (C) line intensity ratio (TC ratio) in test samples with higher AFP concentration. Similarly, in test samples with lower AFP concentrations, binding of the AuNP–antihuman–AFP–antibody to form a sandwich complex at the test line is reduced, thereby causing an overall decrease in the TC ratio.

### AuNP–Antihuman–AFP–Antibody Conjugate Pad Preparation

The antihuman–AFP–antibody was conjugated with AuNPs, following the protocol provided in the gold conjugation kit. The protocol provided by the vendor was used to obtain AuNP–anti-AFP–antibody conjugation. To remove the excess unbound antibodies, a 1:10 dilution of the quencher with water was added up to 10 times the volume of the conjugate mixture and the suspension was centrifuged at 9000 × g for 10 min. The remaining pellet of AuNP–anti-AFP–antibody was resuspended in a solution comprising a 1:10 dilution of quencher with water. The final optical density (OD) was measured using a Spectramax 384 spectrophotometer at 530 nm. The AuNP–anti–AFP conjugate was diluted to 0·35 OD in a conjugate buffer (2 mM borate buffer with 5% sucrose). The conjugate pad was soaked in diluted conjugates for 1 min and oven dried at 37°C for 2 hours, followed by storage at room temperature overnight.

### Test Strip Assembly

The membrane card comprised a polyester film backed with a nitrocellulose layer on top. Striping of the test and control line antibodies (1 mm wide and 3 mm spacing), consisting of antihuman AFP–antibody and antimouse–IgG on the nitrocellulose membrane, was performed using the lateral flow antibody dispenser. Membrane cards were then immediately dried for 2 hours at 37°C in a forced convection oven and stored at room temperature in a humidity-controlled box. The conjugate pad, absorbent pad, and sample pad were then assembled with a 2-mm overlap between each pad. The assembled card was cut using an automated shear cutter to obtain test strips of 5 mm width.

### Testing Protocol

**Figure 2.**
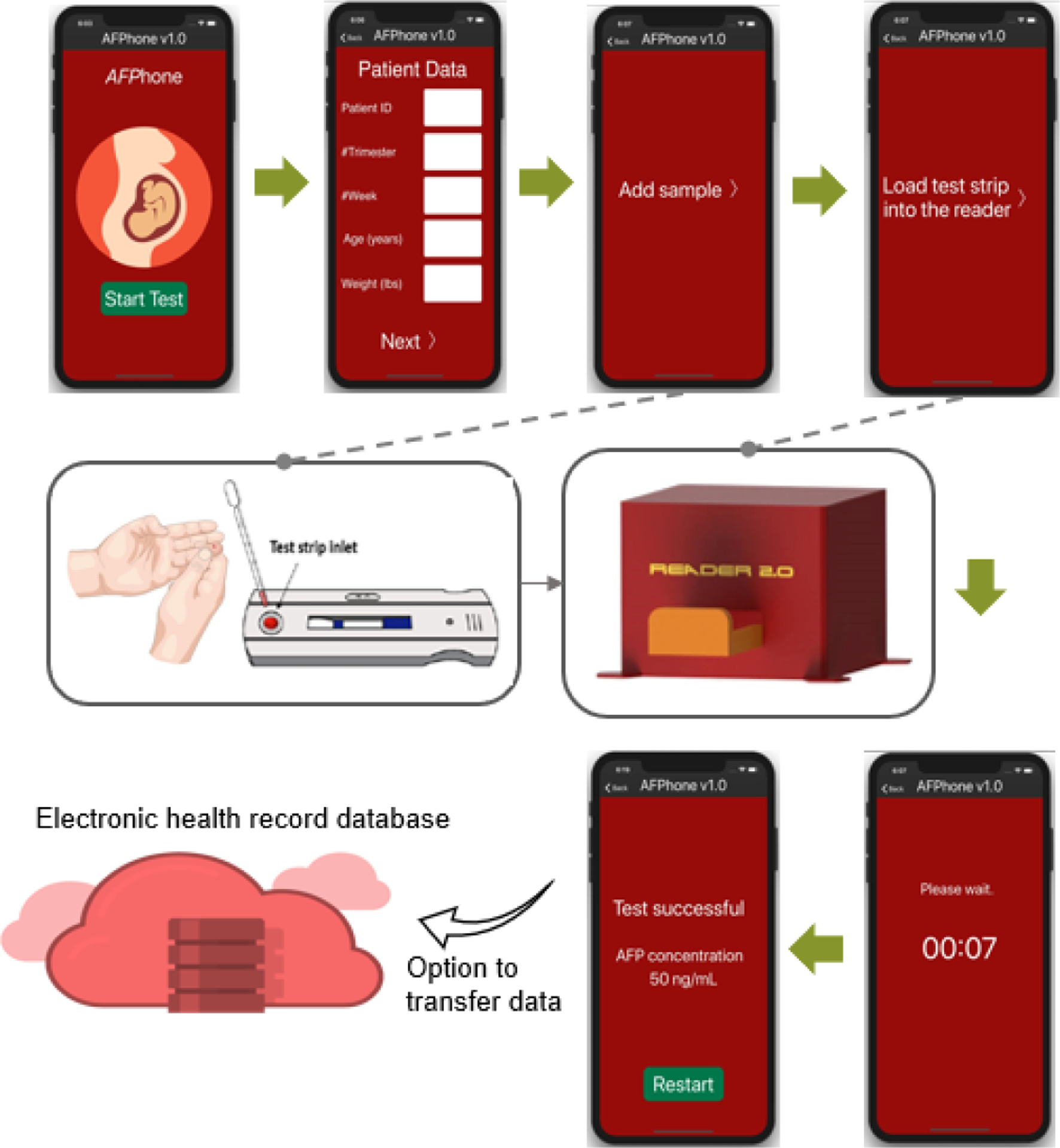
Testing protocol with representative screenshots of the mobile app providing the user with step-by-step instructions.

Figure 2 shows a schematic of the various steps involved in conducting point-of-care AFP testing. The user is guided with step-by-step instructions on the mobile app. Briefly, the user first adds the test sample comprising a mixture of the archived serum or serum-based standards and chase buffer (1×Tris-buffered saline with 1% BSA, 1.5% Tween20, and 0.1% sodium azide) to the test strip to initiate capillary flow within the test strip, which causes the AuNP–antihuman–AFP–antibody conjugates to be released from the conjugate pad. The free AFP in the test sample reacts with the AuNP–antihuman–AFP–antibody and flows downstream to further react with antibodies at the test and control lines. The remaining sample was finally collected in the absorbent pad. The user inserts the test strip into the test strip reader to capture the images of the colorimetric signals with the camera and to analyze via the mobile app to provide the AFP concentrations.

### Preparation of AUDIT AFP Calibrators

The AUDIT AFP calibrators (vials S0-S5) available in a lyophilized form were dissolved in deionized water, based on the instructions provided in the kit to obtain concentrations ranging from 4 to 1034 ng/mL.

### Image Processing

The mobile app performs image processing steps on the captured test strip image to improve the accuracy and detection limit. Details of the image processing approach have been previously reported. Briefly, captured images are cropped and converted into grayscale to extract the local minima of pixel intensities and calculate the TC ratios, which correlate with AFP concentrations.

### Statistical Analysis

All analyses were performed using Excel (Microsoft) and R software (RStudio version 1.1.456, RStudio Inc). TC ratios were compared with AFP concentrations determined by the reference method with nonparametric bootstrap resampling analysis conducted in R.

## Results

### Calibration Curve for AUDIT AFP Calibrators

**Figure 3.**
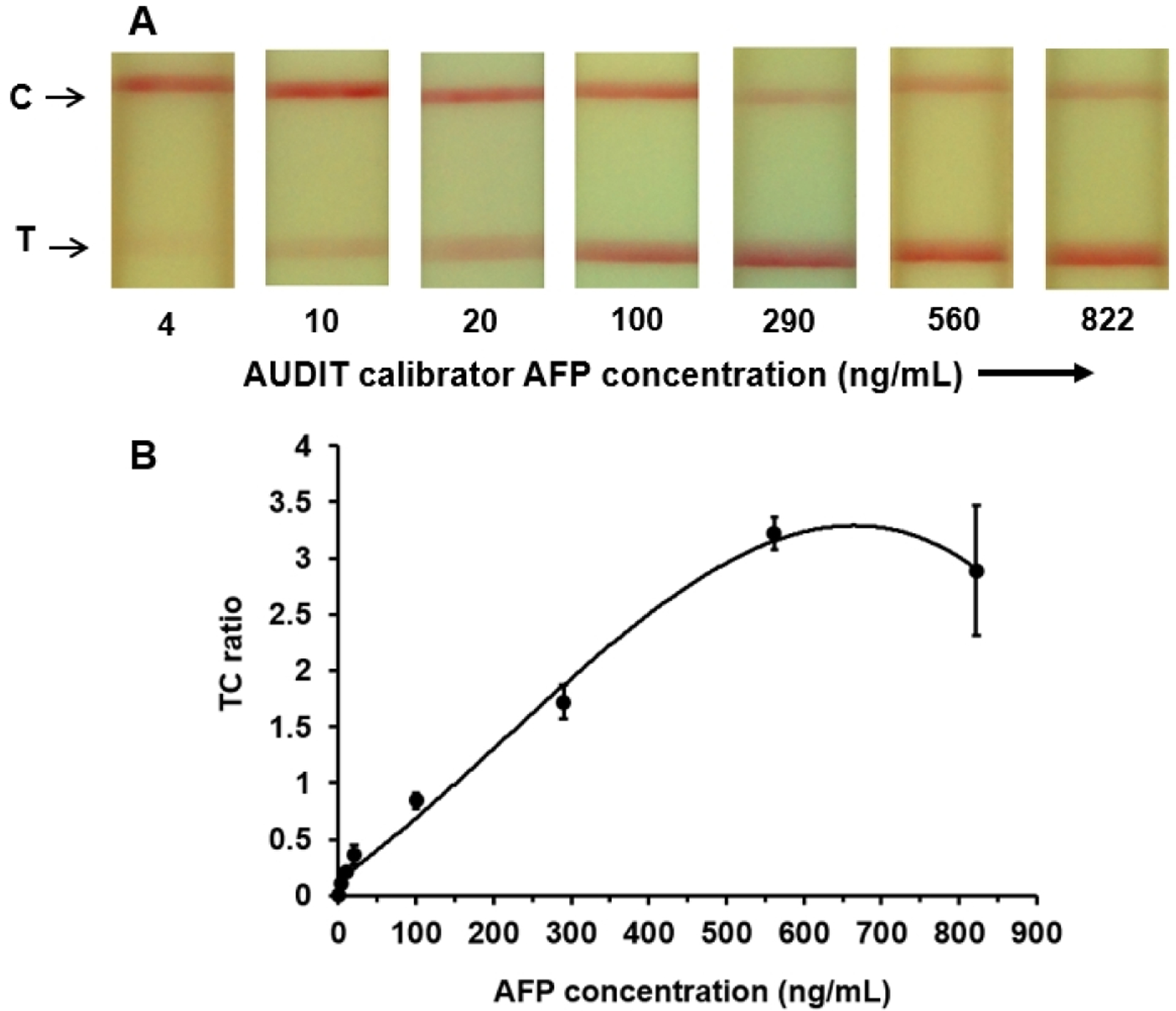
(A) Colorimetric variation of the test and control line regions on the alpha-fetoprotein test strip at various known concentrations of alpha-fetoprotein in AUDIT serum-based calibrators. (B) Calibration curve showing the test and control line intensity ratios of the colorimetric signals at various alpha-fetoprotein concentrations in AUDIT serum-based calibrators. AFP: alpha-fetoprotein; C: control; T: test.

Commercially available AUDIT calibrators were obtained as 5 separate vials labeled A-E, with concentrations ranging from 4 to 1034 ng/mL. Testing was performed simultaneously for each concentration in triplicates. The colorimetric changes in the test and control lines at various known concentrations of the AUDIT calibrators are shown in Figure 3.

The calibration curve shown in Figure 3 demonstrated that the TC ratios were correlated with the AFP concentration in the calibrators. The TC ratio increased with increasing AFP concentrations until approximately 650 ng/mL and then began to decrease beyond the physiological range because of the hook effect [31,32].

### Calibration Curve for Human Serum Samples

**Figure 4.**
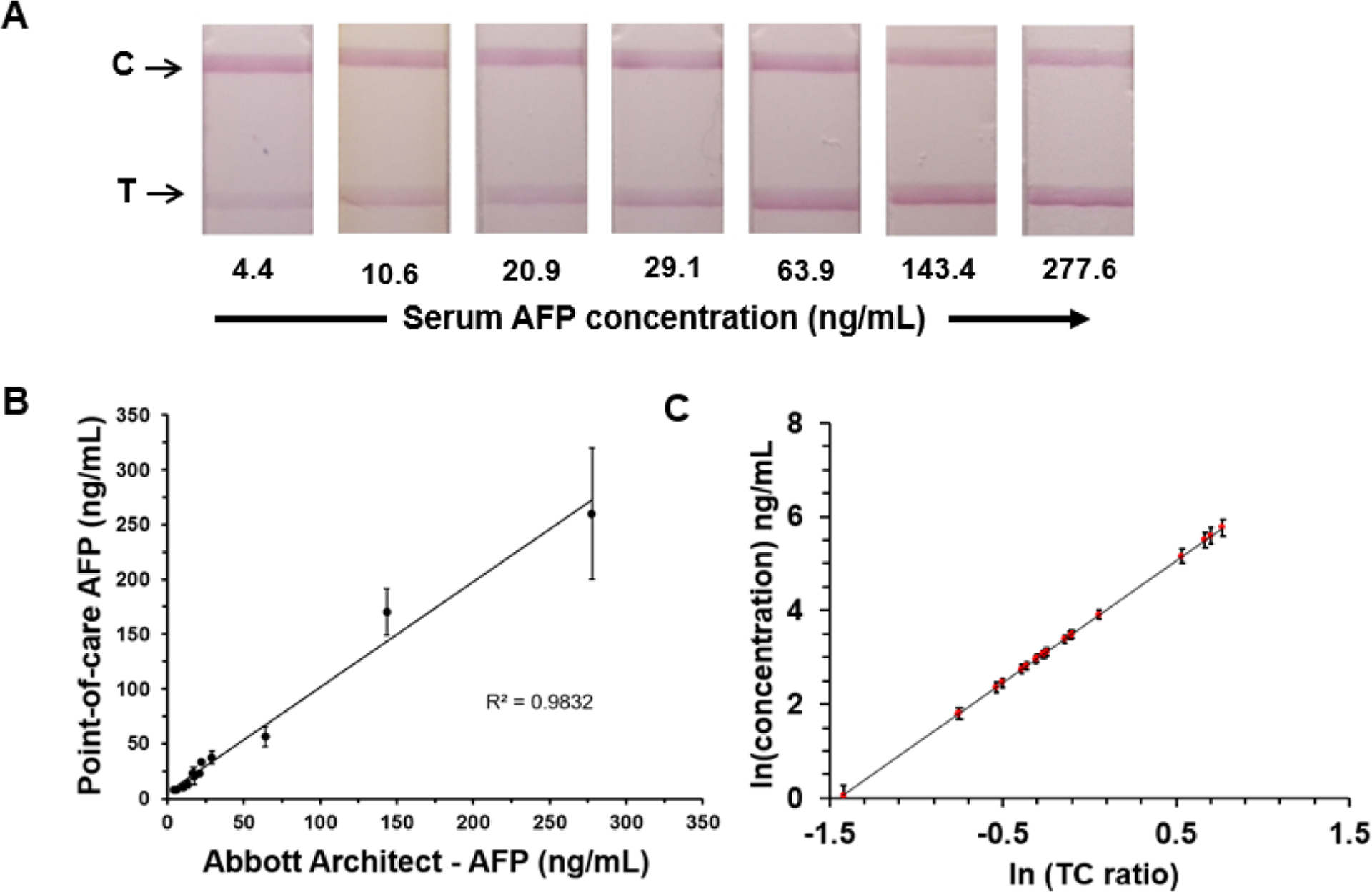
(A) Colorimetric variation of the test and control line regions on the alpha-fetoprotein test strip for serum samples. (B) Correlation plot of serum alpha-fetoprotein concentrations predicted from point-of-care test strip results against the corresponding Abbott ARCHITECT results. (C) Results of bootstrapping to compare bootstrap means of predicted alpha-fetoprotein concentrations and standard errors for the observed test and control line intensity ratio values from a power model using 1000 resampled data sets. AFP: alpha-fetoprotein; TC: test and control line intensity.

The performance of the AFP test strips was further evaluated using archived human serum samples. Serum samples included commercially available serum samples with known AFP concentrations provided by the vendor based on Abbott ARCHITECT CMIA. The colorimetric changes in the test and control lines at various known concentrations of serum samples are presented in Figure 4.

We selected TC data for 8 samples and compared our test strip results with the corresponding reference method (Abbott ARCHITECT) results to obtain an initial calibration curve. This calibration curve was then applied to predict the AFP concentrations of the remaining samples tested on the mobile platform. Figure 4 shows a correlation plot comparing the AFP levels predicted by our point-of-care technology against the corresponding levels provided by the vendor, based on Abbott ARCHITECT testing.

Bootstrap resampling analysis was performed using RStudio with serum AFP concentration results of 20 samples to assess the correlations between TC ratios determined on the point-of-care system with Abbott ARCHITECT–determined AFP concentrations. The bootstrapping function was applied to resample 1000 times, and the resulting correlation coefficients were computed. Figure 4 presents the bootstrapping results to compare the bootstrap means of predicted AFP concentrations and SEs for the observed TC ratio values from a power model, using 1000 resampled data sets. A 95% CI for the correlation coefficient between TC ratios and AFP concentrations of the tested serum samples was also obtained (0.846–0.975). Findings from bootstrap analyses provide quantitative evidence that TC ratios from our point-of-care technology and AFP concentrations of the tested serum samples are highly correlated.

## Discussion

### Principal Findings

In this study, we demonstrated a proof of concept for a sandwich-type immunoassay test strip on our mobile platform for the quantification of AFP concentrations in human serum samples. We determined calibration curves for the AFP assay on a mobile platform with commercially available AUDIT AFP serum-based calibrators and commercially obtained serum samples with known AFP concentrations. The detection range demonstrated in the AUDIT AFP calibrators was 4–650 ng/mL, which covers the range of maternal serum AFP levels observed during pregnancy. The point-of-care AFP assay on the mobile platform was successfully applied to quantify the AFP concentrations in commercially obtained human serum samples with concentrations ranging from 2.2 to 456 ng/mL based on the Abbott ARCHITECT method. On preliminary testing and comparison with Abbott ARCHITECT, a correlation of 0.98 (*P*<.001) was observed with high sensitivity.

### Interpretation of the Findings

On preliminary testing and comparison with Abbott ARCHITECT, a correlation of 0.98 (*P*<.001) was observed with high sensitivity. The detection range of our point-of-care assay covers the range of maternal serum AFP levels observed during pregnancy. The mobile platform for AFP has the capability to quantify AFP within a few minutes without the need for expensive and time-intensive methods. Obstetricians worldwide face the challenge of screening high-risk pregnancies among the overwhelming number of pregnant women presenting to hospitals. Advances in medical technology often widen health disparities, seldom reaching resource-limited populations. State-of-the-art diagnostic equipment is costly, is bulky, and requires sophisticated training for operation and maintenance. Shifting the approach from clinic- and laboratory-dependent care to a mobile platform based on our point-of-care approach will enable translation to resource-limited settings and the global health care market. Mobile devices, which are increasingly ubiquitous even in resource-limited settings, have the capacity to transform clinical care, health services, research, and surveillance [33,34] across populations. An early confirmation of high-risk pregnancies gives parents more time to process the information and learn about early intervention programs including establishing care in a patient-centered medical home, reviewing eligibility for parental financial and psychological support programs. Clinicians can provide parents with unbiased, comprehensive, and culturally sensitive information about congenital birth defects and available services. An early screening is critical to identify and enroll newborns in state-specific early intervention programs as soon as possible to improve the short- and long-term outcomes.

Screening for MSAFP concentrations is a standard of prenatal care to identify pregnancies that may increase the risk of NTDs and some trisomy-affected pregnancies, such as trisomy 21 (Down syndrome) and trisomy 18 (Edwards syndrome). These methods aim to screen for the risk of adverse pregnancy outcomes by quantifying biochemical markers in the maternal serum and, in some cases, also incorporate fetal nuchal translucency measurements obtained via ultrasound. This screen is aimed at identifying pregnancies at a higher risk, so that these patients can be offered diagnostic testing and counseling if required. MSAFP results during pregnancy are usually expressed as multiple of the median (MoM) for each gestational week. MoM values are easy to derive, more stable, and allow for an interlaboratory variation. MSAFP levels that are above 2.0 to 2.5 MoMs are considered abnormal. Using the MSAFP cut-off level of 2 to 2.5 MoM for a given gestational age in singleton pregnancies, the detection rates are approximately 95% for anencephaly and between 70% and 85% for other NTDs [35]. In many cases, high-resolution ultrasound is used in conjunction with MSAFP screening, with detection rates depending on gestational age and the type and severity of the NTD.

MSAFP assessment can also be used to screen for other fetal malformations. Studies have shown that elevated second trimester MSAFP can also indicate gestational age, multiple pregnancies, intrauterine death, or other fetal malformations [36]. High MSAFP levels are also observed in certain types of ovarian germ cell tumors (eg, endodermal sinus tumor and embryonal carcinoma), with levels often greater than 1000 ng/mL, especially with pure endodermal sinus (yolk sac) tumors, in which MSAFP levels are greater than 10,000 ng/mL. MSAFP screening has been shown to play a valuable role in the management of twin pregnancy [37], both in the detection of twins and in the prediction of perinatal outcomes in twin pregnancies.

When MSAFP is elevated, targeted ultrasound examination is offered as the initial diagnostic test, in addition to, or in place of, amniocentesis. The quadruple test, which includes AFP, is performed in the early second trimester, optimally at 15–18 weeks of gestation. Errors in the estimation of gestational age [38,39] are the most common reason for a false-positive result. If the true gestational age is earlier than reported, then AFP MoM values will be falsely interpreted as low. However, in resource-limited settings, even with a high number of false positives, a screening test can identify those in need of an ultrasound; furthermore, the number of false positives can be reduced with simple sequencing during clinical examinations, such as matching the last menstrual period with fundal height assessment where relevant and feasible. The sensitivity of serum AFP screening for NTDs has been shown to significantly improve when the gestational age used for the AFP MoM calculation was verified by ultrasound [40]. There is also evidence that in pregnancies resulting in spontaneous early preterm delivery, MSAFP level at 11–13 weeks’ gestation is higher, and MSAFP measurement improves the prediction of preterm delivery compared with maternal characteristics and obstetric history alone [41]. The mobile app for this test can be designed to include a data collection module to compile maternal age, weight, ethnicity, gestational age, and other relevant patient history. This additional patient information presented along with the AFP results can enable an obstetrician to interpret the test results and make informed decisions regarding any diagnostic and clinical follow-up. The mobile app can also be designed to wirelessly transmit the test result data to a centralized database of laboratories or public health agencies that can be accessed by obstetricians to interpret the test results and make informed decisions regarding any diagnostic and clinical follow-up.

Serum AFP is a widely accepted serum marker for the detection of hepatocellular carcinoma (HCC). Serum AFP is elevated in tumors, including HCC, hepatoblastoma, and nonseminomatous germ cell tumors of the ovary and testis. Most studies report elevated AFP concentrations in approximately 70% of patients with HCC and in 50% to 70% of patients with nonseminomatous testicular tumors. The 5-year survival rate of primary liver cancer is approximately 15%, and the mortality rate is mainly attributed to late diagnosis in many patients, with a high recurrence rate after curative treatment. There is an urgent need for regular screening for patients at risk for HCC to enable an early detection of this tumor or its recurrence. The second model list of essential in vitro diagnostics list (EDL) was recently released by the World Health Organization for detecting, diagnosing, and monitoring a wide array of disease conditions. There is an urgent need for POCT, especially for the diagnostics listed under the first tier in community and health settings without laboratories. The second model list of EDL includes AFP diagnostic testing for the screening of HCC and staging and disease monitoring of germ cell tumors. HCC accounts for 70%−85% of all primary liver cancers and is the ninth leading cause of cancer-related deaths in the United States.

In areas with the highest burden of NTDs, such as India [5,42–44], there is also limited access to centralized laboratories, cold chain, amniocentesis, and ultrasound technology. Such limited access makes a POCT device for screening NTDs especially relevant for these populations. The use of a drop of capillary blood from a finger prick in our approach is minimally invasive, and sample collection is faster compared with venous blood sampling used in a traditional laboratory approach. A study comparing AFP values of capillary and venous blood in 43 participants concluded that there were no significant differences, with a high correlation (*r*=0.995) between the sampling methods [45]. AFP concentrations in men and nonpregnant women vary by age and race but are typically in the range of 0–40 ng/mL. MSAFP levels in pregnancy begin to increase beginning around 14 weeks of gestation until approximately 32 weeks. Between weeks 15 and 20, MSAFP levels mostly range between 10 and 150 ng/mL [46]. In adults, serum AFP levels greater than 200 ng/mL in patients with liver cirrhosis are a strong indicator of HCC.

Our preliminary work presented here is a quantitative, point-of-care lateral flow immunoassay–based screening test for the quantification of serum AFP concentrations. This technology will enable a rapid screening of high-risk pregnancies and enable physicians to make informed decisions, especially in resource-limited settings with limited access to diagnostic laboratories. The ACOG recommends integrated or sequential screening tests with high detection rates earlier in pregnancy, which can provide patients with diagnostic options to consider. The findings suggest that at the current stage of development, this technology can play a significant role as a screening tool for high-risk pregnancies to assess whether further diagnostic testing may be needed.

### Limitations

This study has some limitations. There were limited number of test samples used in this study. In addition, there is a need for validation with larger samples in our future work and a more comprehensive evaluation of the diagnostic performance. In future studies, we plan to optimize the performance of the AFP point-of-care assay on a mobile platform using whole blood in human validation studies among a greater number of participants with a broader range of AFP concentrations to improve the calibration curve and conduct an extensive evaluation of diagnostic performance.

### Conclusions

In conclusion, we developed and performed a preliminary testing of a point-of-care screening test on our mobile platform for the detection of serum AFP levels from a drop of serum sample within a few minutes. On the basis of preliminary testing results, the AFP screening test on the mobile platform reported in this study will pave the way for our future work focused on developing a point-of-care mobile device–enabled quad-screen test in real time in clinical and field settings. State-of-the-art diagnostic equipment is costly, is bulky, and requires sophisticated training for operation and maintenance. Shifting the approach from clinic- and laboratory-dependent care to a mobile platform based on our point-of-care approach will enable translation to resource-limited settings and the global health care market. Screening for high-risk pregnancies will enable physicians to make informed decisions on whether further diagnostic testing, such as ultrasound and amniocentesis, should be considered. Prenatal NTD detection also informs decisions about the optimal time, route, and site of delivery. Our future work will focus on conducting appropriately powered diagnostic test accuracy studies with maternal serum samples and developing a multiplexed assay to include hCG and unconjugated estriol as a part of the triple screening test. An early confirmation of high-risk pregnancies provides parents more time to process the information and learn about early intervention programs, including establishing care in a patient-centered medical home, reviewing eligibility for parental financial and psychological support programs. An early screening is critical to identify and enroll newborns in state-specific early intervention programs as soon as possible to improve short- and long-term outcomes. Overall, the preliminary results reported in this work serve as a foundation for our future research focused on developing a quad-screen POCT to enable (1) screening for high-risk pregnancies within a few minutes at the point of care even in remote areas; (2) identification of patients who might need continued health care advice and counseling; (3) planning for enrolling newborns in state-specific early intervention programs as soon as possible to improve short- and long-term outcomes; and (4) development of surveillance tools for birth defects.

## Abbreviations

ACOG: American College of Obstetricians and Gynecologists
AFP: alpha-fetoprotein
AuNP: gold nanoparticle
BSA: bovine serum albumin
CMIA: chemiluminescent magnetic microparticle immunoassay
EDL: essential in vitro diagnostics list
HCC: hepatocellular carcinoma
hCG: human chorionic gonadotropin
IgG: immunoglobulin G
MoM: multiple of the median
MSAFP: maternal serum alpha-fetoprotein
NTD: neural tube defect
OD: optical density
POCT: point-of-care testing
TC: test and control line intensity
